# Pharmacotherapy of Kienböck Disease and the Carpal Avascular Necrosis Family: A Narrative Review with a Four-Axis Translation Framework

**DOI:** 10.3390/medicines13030027

**Published:** 2026-09-01

**Authors:** Dimitar Tenev, Benedikt Hochbein, Georgi Enev, Georgi Raykov, Nikolay Cherkezov, Jakob Adolf, Nikolay Dimitrov

**Affiliations:** 1 USBALO—Boycho Boychev, Medical University Sofia, 1614 Sofia, Bulgaria; 2 CMSC, Charité—Universitätsmedizin Berlin, 10117 Berlin, Germany; 3 Department of Consultation-Liaison Psychiatry and Psychosomatic Medicine, University Hospital Zurich, University of Zurich, 8091 Zurich, Switzerland; 4 Clinic of Orthopaedics and Traumatology, Sonnenhofspital, 3006 Bern, Switzerland

**Keywords:** drug repurposing, pharmacotherapy, osteonecrosis, clinical translational research, iloprost, bisphosphonate, teriparatide, Kienböck disease, lunate, basket trial

## Abstract

**Background/Objectives:** Kienböck disease (idiopathic avascular necrosis of the lunate) is treated almost exclusively surgically; its pharmacological evidence base comprises fewer than five primary publications and ~20 patient-level observations. Femoral-head, jaw, and knee osteonecrosis have decades of evidence, including randomised trials. We ask whether extra-carpal evidence can be translated to Kienböck disease and the wider Carpal Avascular Necrosis Family (lunate, scaphoid/Preiser, capitate, hamate, triquetrum). **Methods:** We conducted a narrative review reported per SANRA, searching PubMed, CrossRef, Google Scholar and three specialty registries from inception to 2026 across eleven drug classes and eleven skeletal sites. All references were independently verified against primary sources (PubMed/CrossRef). No Kienböck-specific randomised or controlled data exist; all conclusions therefore rest on indirect, extra-carpal evidence. **Results:** Three pathways (subchondral insufficiency fracture, microvascular ischaemia, hypercoagulability) predict drug-class plausibility differently by site. Iloprost is the candidate most consistently aligned across the translation axes, though no direct lunate data exist; teriparatide provides the only randomised evidence suggesting reversal of established osteonecrosis at any site (medication-related osteonecrosis of the jaw). The lunate sits within a coherent carpal AVN family sharing end-arterial supply, Lichtman-grade trajectories, and a near-identical evidence vacuum. **Conclusions:** A Four-Axis Translation Framework (mechanistic alignment, anatomic concordance, Lichtman-stage applicability, lunate-specific risk–benefit geometry) is proposed to evaluate which extra-carpal evidence is admissible at the lunate; with the carpal AVN family construct, these are the review’s two novel contributions.

## 1. Introduction

Kienböck disease, an idiopathic avascular necrosis of the lunate first described by Kienböck in 1910, burdens working-age, dominant-hand-using adults [1,2,3,4]. Annual incidence is below 1 per 100,000 [1,4]; typical patients are manual workers, athletes or musicians in their third to fifth decades of life. Untreated, the disease progresses through four Lichtman stages from bone-marrow oedema to fragmentation, carpal collapse, and pancarpal arthrosis [2,3].

Management is overwhelmingly surgical and stratified by Lichtman stage, with conservative immobilisation retained for the earliest disease and as salvage for surgically unfit patients [5,6,7,8]. Later stages draw on joint-levelling osteotomies, vascularised bone grafts, carpal arthrodeses, proximal-row carpectomy and, at salvage, total wrist arthroplasty [2,3,4,9,10]. The Lichtman 2017 algorithm [3] integrates osseous stage (Lichtman), perfusion (Schmitt) and arthroscopic cartilage type (Bain & Begg [11,12]; see Section 5.3). Innes and Strauch’s 2010 systematic review reached the field-defining verdict that no procedure is superior to any other or to natural history [13]; Aspenberg proposed an earlier biological rationale of revascularisation-driven trabecular collapse [14]. No algorithm integrates a drug-based primary or adjunctive arm.

The Kienböck pharmacological evidence base is categorically thin: fewer than five primary publications and ~20 patient-level observations, namely three Kienböck patients in Agarwala’s non-femoral AVN bisphosphonate cohort [15], eight in Mazhar’s local-deferoxamine pilot [16], five in a registered autologous MSC phase-I series [17], two single-patient HBOT reports [18,19], and Ogawa’s combined-modality series [20]. There are no RCTs, controlled cohorts, or Lichtman-stage-stratified drug studies; the Innes–Strauch verdict extends into pharmacology [13]. This contrasts with the mainstream AVN sites: two opposing femoral-head bisphosphonate RCTs [21,22], multiple meta-analyses [23], iloprost cohorts (Section 5), a mature MRONJ consensus [24], the only RCT proving pharmacological reversal of established osteonecrosis (teriparatide for MRONJ) [25], and the SPONK-as-SIF reframe with at least one randomised bisphosphonate trial [26].

This narrative review, with selective transparency enhancements and search reported per SANRA [27], appraises extra-carpal pharmacotherapy evidence translatable to Kienböck disease, positioning the lunate within a carpal AVN family (scaphoid/Preiser [28], capitate, hamate [29], triquetrum) sharing end-arterial supply, comparable Lichtman trajectories and a near-identical evidence vacuum. Surgical interventions are reviewed elsewhere [2,3,9,10,13,30]. Two novel contributions are advanced: the Carpal Avascular Necrosis Family, formalised as a mechanistically coherent basket-trial population, and a Four-Axis Translation Framework operationalising admissibility of extra-carpal evidence. Written for a pharmacology and clinical–translational-research readership, the review treats Kienböck disease as a model thin-evidence indication in which extrapolation from extra-carpal pharmacological evidence to the lunate is the central drug-repositioning question.

## 2. Materials and Methods

### 2.1. Review Design and Reporting Framework

This is a narrative review with selective transparency enhancements, covering pharmacological treatment of Kienböck disease and the wider osteonecrosis (AVN) and bone-marrow-oedema literature from which its conservative-management evidence is extrapolated. Systematic review/meta-analysis was rejected at the planning stage: the Kienböck-specific base is too sparse (Section 1), and the unifying question (*what should the wrist clinician prescribe today, on what extra-carpal evidence?*) is integrative rather than enumerative.

Reporting adheres to SANRA [27]; we report the *a priori* review question, search strategy, date range, eligibility, verification, synthesis approach, and explicit limitations, following scoping- and narrative-review conventions without formal adoption of either. Screening counts and risk-of-bias scoring (PRISMA artefacts) are omitted, and the review was not registered in PROSPERO.

### 2.2. Search Strategy

We searched PubMed, CrossRef and Google Scholar, supplemented by the Cochrane Library, Thieme-Connect, the AWMF Leitlinienregister and Italian/Russian/Bulgarian specialty journals where citation trails led (inception to 2026). Ten thematic packs crossed eleven drug classes with eleven skeletal sites and applied study-type filters. Reference lists of all included reviews and consensus statements were hand-searched. The final search was completed on 1 July 2026. Two authors (D.T., J.A.) independently screened and selected studies, with third-author adjudication (N.D.).

### 2.3. Eligibility Criteria

We included primary clinical studies, systematic reviews, meta-analyses, consensus guidelines and mechanistic studies of any pharmacological intervention for non-traumatic osteonecrosis or bone-marrow oedema at any skeletal site, provided the evidence could be reasoned to Kienböck directly or by mechanistic/site-stratified extrapolation. We excluded purely surgical series without a pharmacological arm, post-traumatic AVN, animal studies without clinical correlate, and herbal/complementary interventions. Languages included English, with German, Italian, and Russian/Bulgarian sources where directly relevant and retrievable.

### 2.4. Synthesis and Quality-of-Evidence Tagging

Findings are synthesised narratively by drug class and, within each class, by skeletal site; the drug × site matrix (Table 1) and the four-axis framework (Table 2) are the central reference tools. Full GRADE [31] was judged over-engineered; we instead apply a modified GRADE-style QoE tag (Very Low to High) by integrated judgement of study design, sample size, mechanistic alignment and site-indirectness. Each block began from the OCEBM level of its best available study and was downgraded one tier each for serious site-indirectness, sparse or uncontrolled data, and inconsistency; no block reached above Low without Kienböck-specific controlled data. Only the iloprost recommendation for Lichtman I–II with confirmed MRI BME, and as surgical adjunct in selected Stage IIIA cases (Section 5.7 and Section 9.3), rises to Low; none reaches Moderate or High.

### 2.5. Limitations and Risk-of-Bias Considerations

Structural biases of the underlying evidence base are as follows: (i) Vienna single-centre dominance in the iloprost-AVN literature, inherited by the Zippelius 2022 meta-analysis [32]; (ii) Agarwala investigator-allegiance across the bisphosphonate-AVN programme [33,34,35,36]; (iii) publication bias in the small-series literature of positive single-arm reports; (iv) staging confounding, as pre-2010 cohorts predate the modern Lichtman algorithm [3]; (v) selective short-term-outcome reporting; and (vi) indication bias in retrospective comparatives [37].

## 3. Pathophysiology of Osteonecrosis: Cross-Site Mechanisms

### 3.1. Three Convergent Mechanistic Pathways

Osteonecrosis is the result of the convergence of three partially overlapping pathways: subchondral insufficiency fracture (SIF), microvascular ischaemia, and hypercoagulability/thromboembolism [33,34]. Their relative contribution varies with site, comorbidity and timing, and contemporary models of Kienböck disease (KD), femoral-head osteonecrosis (ONFH) and SPONK adopt this multifactorial logic [35,36,38].

The SIF pathway was first articulated for SPONK by Yamamoto and Bullough’s 2000 histopathology [39], which reframed the lesion as a subchondral fatigue fracture with secondary post-fracture trabecular necrosis; the Lichtman reformulation treats lunate fragmentation in stages IIIB–IV as a comparable microfracture-driven cascade [3], and femoral-head SIF is histologically indistinguishable from late ONFH [21,40].

The microvascular ischaemia pathway retains primacy in classic ONFH, where reliance on terminal medial femoral circumflex branches creates a corticosteroid-, embolic-, and trauma-susceptible watershed [41]; the lunate parallels this via Gelberman’s Type I single-intraosseous-vessel pattern, present in 7–26% of specimens [42].

The hypercoagulability/thromboembolic pathway, championed by Glueck for ONFH [43], implicates heritable thrombophilias (factor V Leiden, protein C/S deficiency, MTHFR polymorphisms, elevated PAI-1) [22] in subchondral microvascular occlusion. The KD evidence is sparse, but the pathway underlies enoxaparin trials in ONFH and screening in bilateral or early-onset Kienböck.

### 3.2. Site-Specific Anatomical Determinants

Anatomic context determines the dominant pathway. The femoral head is large, end-arterially supplied, and weight-bearing, favouring convergence of overload, ischaemia, and, in steroid/thrombophilia hosts, thromboembolism [44]. The lunate is small, intra-articular and synovial-fluid-bathed yet is the central load-transmitting keystone of the proximal carpal row: the radiolunate articulation transmits approximately 30–50% of axial radial-side load, negative ulnar variance amplifies proximal-pole compression, and local transmission, not whole-limb loading, is the biomechanical substrate of the SIF arm [42]. The medial femoral condyle (SPONK) is intra-articular but heavily loaded, with meniscal extrusion driving SIF [35]; talus, scaphoid (Preiser) and metatarsal head (Freiberg) share watershed architecture.

The jaw in MRONJ is the conspicuous outlier: it has high baseline remodelling, unique oral-microbiome exposure via the periodontal sulcus, and a thin overlying mucosa whose breach permits bacterial colonisation of suppressed bone.

### 3.3. Molecular Determinants

Five molecular axes underpin Section 4, Section 5 and Section 6. RANK/RANKL/OPG governs osteoclast differentiation [45]: bisphosphonates disrupt mevalonate-pathway prenylation, denosumab sequesters RANKL (Section 6.2). Wnt/sclerostin is the canonical anabolic axis [46]: teriparatide and abaloparatide drive PTH-pulse Wnt activation; romosozumab releases the sclerostin brake. HIF-1α-mediated angiogenesis couples vascular ingrowth to osteogenesis [47]: iron chelators prevent HIF-1α prolyl hydroxylation, the rationale for deferoxamine in bone AVN. PPARγ-driven marrow adipogenesis is the substrate of glucocorticoid-induced AVN; statins partially suppress it and restore osteoblastogenesis (Section 6.3). Prostacyclin/IP-receptor pharmacology anchors Section 5 [48]: iloprost engages the Gs-coupled IP receptor on vascular smooth muscle, platelets, endothelium, and, at lower affinity, bone cells, raising cAMP.

### 3.4. Mechanism–Drug Matching

Mechanistic stratification scaffolds drug–disease matching: SIF-dominated lesions call for anti-resorptives to slow remodelling of necrotic trabeculae during creeping substitution [49,50,51]; ischaemia-dominated lesions rationalise vasoactive and rheologic agents (iloprost, HBOT) [48,52]; lesions with substantial viable bone awaiting rebuilding invite anabolic therapy [53,54]; and multi-pathway disease, the realistic majority, justifies sequential or combination regimens [55,56]. Articulated for ONFH by Mont and Hernigou [57,58] and recently adapted to KD, this approach supplies the central translational argument [36,55].

### 3.5. The Bisphosphonate/MRONJ Paradox Resolved

The most common objection to bisphosphonates in KD is MRONJ, but the paradox resolves mechanistically. The AAOMS-2022 position paper [24] defines MRONJ clinically (anti-resorptive/antiangiogenic exposure; exposed or fistula-probable maxillofacial bone persisting >8 weeks; no jaw irradiation or metastasis), a case definition, not an etiologic model. Three converging factors explain jaw localisation, none operating at the lunate [59,60]: high local bone turnover (alveolar bone formation approximately 3–5× cortical long bone [61]); oral-microbiome breach (the lunate is sealed in sterile synovial fluid); and thin vulnerable mucosa (the lunate is enveloped in robust ligamentous and chondral tissue). In murine models [62,63], bisphosphonate-loaded femora developed osteonecrosis-like lesions only when challenged with *P. gingivalis*, establishing microbial breach, not anatomic location, as the necessary co-factor; Reid 2007 first proposed the soft-tissue-toxicity hypothesis [64]. Procedural breach (intercarpal arthroscopy, core decompression, salvage PRC, or peri-procedural deep infection) in a bisphosphonate-loaded patient is therefore the only conceptually live MRONJ-analogous risk, warranting pre-operative dental review and antibiotic-prophylaxis re-assessment.

### 3.6. Aspenberg’s Monkey–Lunate Observation and the Timing Hypothesis

Aspenberg’s 1994 cynomolgus-monkey study, in which experimentally devascularised lunates revascularised with transient structural weakening and partial collapse, is the foundational observation for a remodelling-mediated carpal-bone collapse mechanism [14]. It remains suggestive: lunates were acutely excised and reimplanted rather than being chronically ischaemic, and the imbalance unfolded over weeks rather than over the months-to-years of natural-history Kienböck. The corollary *timing hypothesis,* that anti-resorptive therapy during revascularisation may preserve trabecular architecture long enough for anabolic replacement, supplies the rational basis for bisphosphonate timing (Section 4); the Huang 2013 canine lunatomalacia model [65] remains undeployed for drug screening.

## 4. Bisphosphonates

Bisphosphonates carry the largest evidence base of any pharmacotherapy class in non-traumatic osteonecrosis, alongside a notable site-specific paradox, and are the only class for which prospective Kienböck patient-level data exist [15].

### 4.1. Pharmacology

Bisphosphonates are non-hydrolysable P-C-P pyrophosphate analogues that anchor to hydroxyapatite. Aminobisphosphonates (alendronate, risedronate, ibandronate, zoledronate) inhibit farnesyl-pyrophosphate synthase and disrupt prenylation of the small GTPases needed for osteoclast cytoskeletal integrity, inducing osteoclast apoptosis [42,49,50]. Non-nitrogen agents (etidronate, clodronate) act via cytotoxic ATP analogues and are obsolete in AVN. Relative potency spans five orders of magnitude (alendronate 1× to zoledronate 100–1000×) [50]. Oral bioavailability is 0.6–1% and is halved by food, calcium, or coffee; IV bioavailability is 100%. About half of the absorbed drug is taken up at high-turnover sites, with the remainder renally excreted unchanged [44]. Terminal skeletal half-life runs to years. The proposed disease-modifying mechanism in osteonecrosis is preservation of the necrotic trabecular scaffold during post-ischaemic resorption, with secondary reduction in BME and intraosseous pressure.

### 4.2. Femoral-Head AVN: The Lai Versus Chen Reconciliation

The most-cited positive trial is Lai 2005: 40 patients (54 hips) with Steinberg IIC/IIIC ONFH randomised to 25 weeks of alendronate 70 mg/week versus no treatment, with collapse in 2/29 alendronate versus 19/25 controls at ≥24 months (*p* < 0.001) [22]. The Agarwala observational programme reports hip-arthroplasty avoidance rates of 80–87% in pre-collapse disease at 8, 10, and 20 years [33,34,35,36].

The counterweight is Chen 2012, a multicentre double-blind placebo-controlled trial of 65 hips with Steinberg IIC/IIIC over 104 weeks of alendronate 70 mg/week versus placebo, showing no difference across collapse, time to THA (4/32 vs. 5/33, *p* = 0.837), Harris Hip Score, and SF-36 [21]. Meta-analyses (Yuan 2016 [23], Li 2018 [66]) are dominated by the Chen outlier: favourable but heterogeneous effects pre-collapse, no consistent arthroplasty-conversion benefit, wide confidence intervals [23]. The Zhai 2024 network meta-analysis ranks bisphosphonates high for early disease without superiority for arthroplasty avoidance [52]; Mont 2020 classifies them as investigational [57].

**Reconciliation hypothesis (speculative).** The most credible reading is that the Lai-Chen discrepancy reflects lesion-size and stage-mix selection: Lai enrolled within a narrower pre-collapse window with the trabecular framework still mechanically intact, whereas Chen recruited more large Steinberg type-C lesions in which subchondral fate was already determined [67]. The lesson for Kienböck trial design is stage discipline: specify Lichtman stage strictly, treat lesion volume as a stratification covariate, and pre-define a Lichtman I–II pre-collapse population. The femoral-head inflexion point at which anti-resorptive efficacy is lost (subchondral fracture, ≈ARCO III) corresponds, in the wrist, to Lichtman Stage IIIA (lunate collapse with preserved carpal height and alignment); pharmacotherapy cannot reverse established collapse, so from IIIA onward it is at most an adjunct to a surgical pathway, and the reconciliation argument bounds the pharmacotherapy window at Lichtman II.

### 4.3. Knee SPONK: The Closest Comparable

SPONK (reinterpreted as subchondral insufficiency fracture with secondary distal necrosis) is the closest extra-carpal model for Kienböck: a small intra-articular bone region with mechanical-vascular failure and a pre-collapse therapeutic window. The Jureus 2012 prospective alendronate case series (*n* = 17) [68] reported 59% radiographic recovery and 12% surgical conversion versus 25%/32% in untreated historical controls (Jureus 2013 [69]). The same group later showed that lesion size > 40% of AP condylar width predicts near-certain progression [68,69]. Kraenzlin 2010 reported an 80% VAS reduction at six months on combined IV pamidronate plus oral alendronate [70]. The negative anchor is Meier 2014, a randomised double-blind placebo-controlled trial of IV ibandronate in 30 SPONK patients in which only CTX fell while pain, function and imaging matched placebo [26]. Structural plausibility is not proof of efficacy, and the only Level-I trial in the structurally most lunate-like AVN site was null.

### 4.4. The MRONJ Paradox

MRONJ is the counterweight to the claim that bisphosphonates treat osteonecrosis. At oncological dosing (zoledronate 4 mg IV monthly), the incidence is 1–8%, whereas at osteoporotic dosing it falls to 0.001–0.06% per patient-year and about 0.15% after dental extraction [24,60]. The three proposed mechanisms (site-specific alveolar accumulation of legacy bisphosphonate, oral microbial drive, and direct mucosal keratinocyte toxicity) are resolved in Section 3.5; none applies to the lunate, which has no mucosal exposure, no extraction-style alveolus and no resident microflora. The lunate sits structurally on the femoral head side of bisphosphonate risk geometry, not the jaw side. *The one residual MRONJ-analogous risk, procedural breach in a bisphosphonate-loaded patient, is developed in Section 3.5* [62,63,64].

### 4.5. Lunate Kienböck: What Exists?

The dedicated Kienböck bisphosphonate evidence base consists of one paper. Agarwala and Vijayvargiya 2019 [15] enrolled 18 patients with non-femoral AVN in a prospective open-label cohort on a single IV zoledronate 5 mg infusion plus oral alendronate 70 mg/week for one year plus calcium 500 mg/day and vitamin D 400 IU/day; three had Lichtman Stage II Kienböck. Two showed complete BME resolution on MRI by 12 months without radiographic collapse and needed no surgery; the third responded initially but progressed to stage III by 14 months and underwent Modified Graner II at 19 months, the only patient in the 18-case cohort to require surgery [15]. This is consistent with the Aspenberg revascularisation-collapse hypothesis (Section 3.6) [14]. Roth and Maus 2022 and Salva-Coll 2023 extend the bisphosphonate paradigm narratively to carpal sites but cite no further Kienböck-specific primary data [30,71].

### 4.6. Practical Recommendations, Adverse Events, Drug Interactions, and Evidence Gaps

**Position relative to surgical standard of care.** Bisphosphonate use in Kienböck is an adjunct or alternative to surgical management, not a substitute: the surgical pathway (RSO, CSO, VBG; PRC/4CF/TWA at salvage) remains primary from Lichtman IIIA onward, and a pharmacological adjunct is appropriate only at Lichtman I–II or for patients unfit for or declining surgery.

Categorisation by stage (all GRADE Very Low): Stage I–II, adjunct to immobilisation and vitamin D repletion based on the Lai 2005 analogy [22] and the Agarwala BME-resolution signal [15], with the combination protocol under explicit off-label consent at Stage II [15]; Stage IIIA, surgery is primary, with any adjunct resting on mechanistic plausibility only (Section 4.2); Stage IIIB/IV, unsupported.

**Dosing, investigational and extrapolated (three Kienböck patients only): oral alendronate 70 mg weekly ± single IV zoledronate 5 mg at initiation, plus calcium 500–1000 mg/d and vitamin D 400–800 IU/d [15,34]; duration: 1 year per Agarwala. GRADE: Very Low.**

**Adverse events:** Oral N-bisphosphonates cause upper-GI upset and oesophagitis, mitigated by upright administration. The first IV zoledronate dose produces an acute-phase reaction in 30–50%, partially preventable by paracetamol plus vitamin-D repletion. Atypical femoral fracture is the class-specific long-exposure concern (risk rising with exposure ≥ 3 yr and falling quickly after discontinuation [72,73]); the Salamah 2024 systematic review of teriparatide-driven AFF salvage [74] supports an anabolic-rescue pathway. MRONJ at osteoporotic dosing is <0.1% and biologically implausible at the lunate (Section 3.5). Zoledronate is contraindicated below eGFR 35 mL/min. Drug *interactions. Oral bisphosphonate absorption falls by ≥60% when co-ingested with* calcium, iron, magnesium, antacids, dairy, or coffee, mandating a ≥30 min separation interval; PPIs and H2-antagonists impair dissolution. *Additive* MRONJ risk arises with corticosteroids, antiangiogenics and methotrexate/biologic DMARDs. Nephrotoxins (NSAIDs, iodinated contrast, ACE-i/ARB, aminoglycosides) amplify zoledronate-associated AKI, and loop diuretics may precipitate symptomatic hypocalcaemia. Warfarin and DOACs show no meaningful interaction.

**Evidence gaps.** There is no Kienböck-specific RCT for any bisphosphonate; no Lichtman-stratified data beyond Agarwala 2019 (*n* = 3); and no validation of MRI BME-resolution as a surrogate for long-term wrist function or salvage avoidance [15,21,22,26].

### 4.7. Pregnancy, Lactation, and Perioperative Holiday

Because the Kienböck population includes many women of reproductive age and N-bisphosphonates persist in bone for years, pre-conception counselling, effective contraception during treatment and for at least 12 months after oral therapy, and deferral of bisphosphonate therapy in planned pregnancy and lactation are warranted before off-label use [75,76,77,78].

Whether to interrupt N-bisphosphonates before wrist surgery is unresolved. A holiday clears plasma drug within two weeks, but not the years-accumulated skeletal burden, so routine interruption is not justified for elective joint-levelling surgery. For higher-risk salvage procedures, pre-operative dental review and a short interval after the last IV dose are reasonable by extrapolation from the AAOMS 2022 framework [24], though urgent salvage surgery should not be delayed.

## 5. Iloprost and Vasoactive Agents

### 5.1. Pharmacology and Rationale

Iloprost is a chemically stable synthetic prostacyclin (PGI_2_) analogue, administered as an IV infusion over five consecutive days at ~20 µg daily, titrated to body weight and tolerability [32,79]. Licensed indications are pulmonary arterial hypertension and severe Buerger disease/critical limb ischaemia; off-label use in BME and early AVN spans two decades [32,80].

It is a high-affinity agonist at the Gs-coupled prostacyclin (IP) receptor (Section 3.3) [48], and cross-reacts weakly with EP1/EP3/EP4, of which EP4 has documented preclinical bone-anabolic effects. The IP/EP–cAMP cascade produces four clinical effects: microvascular vasodilation, platelet-aggregation inhibition, endothelial anti-inflammatory action, and modest enhancement of endogenous fibrinolysis [32,80]. Each maps onto a pathway implicated in the early Kienböck cascade: venous congestion, marrow oedema, microthrombosis and subchondral ischaemia [36].

**Pharmacokinetics: The IV plasma half-life is approximately 20–30 min, with elimination essentially complete within 1–2 h; clearance is dominated by non-CYP β-oxidation without clinically relevant accumulation. Per SmPC [81], dosing is body-weight-titrated from ~0.5 to 2 ng/kg/min by tolerability.**

### 5.2. Mechanistic Suitability for Early Kienböck

**Lichtman Stage I (BME-only, normal radiographs) and Stage II (sclerosis without lunate collapse) constitute the *true* pre-collapse phase**, in which MRI signal change is dominated by BME and gross lunate architecture is preserved and cartilage involvement is minimal [36]. Stage IIIA (Lichtman 2010/2017 [2,3]: lunate collapse with preserved carpal alignment and normal carpal height) is post-collapse but pre-carpal-arthrosis, sitting at the collapse boundary defined in Section 4.2 where anti-resorptive efficacy is lost, while residual BME and a persistent microvascular substrate often remain tractable as an adjunct to joint-preserving surgery, not a substitute. Every mechanism that iloprost addresses maps directly onto Lichtman I–II pathophysiology, where the drug is a *candidate for prospective investigation*, and onto the residual BME/microvascular component of Stage IIIA, where it serves as a *surgical adjunct*.

### 5.3. Arthroscopic Functional Articular Surface Assessment: The Bain and Begg Classification

Without a cartilage axis, “Lichtman I–IIIA without architectural collapse” cannot be operationalised as a trial entry criterion, and the recommendations of Section 5.7 and Section 8.5 cannot be linked to an enrolment definition.

**The Bain and Begg classification [11], described arthroscopically in 2006** and operationally formalised by Bain and Durrant [12], grades the wrist by the number of non-functional articular surfaces among four: the lunate facet of the radius, the proximal and distal lunate surfaces, and the proximal capitate; a functional surface has normal cartilage over hard subchondral bone, while a non-functional surface has extensive fibrillation, fissuring, articular loss, or fracture.

**In the Lichtman/Pientka/Bain 2017 four-type collapse scheme [3], Type 1 (both lunate articular surfaces intact; Bain 0–1) is the prime joint-preserving and pharmacotherapy population; Type 2A (proximal surface non-functional, distal intact; Bain 1–2A) directs proximal-pole reconstruction with a defensible role for pharmacological adjuncts; Type 2B (distal surface non-functional, typically a coronal lunate fracture; Bain 2B) directs PRC or partial fusion and is no longer mechanism-aligned; and Type 3 (both surfaces non-functional; Bain 3–4) is a salvage situation in which pharmacotherapy is not indicated.**

**Pharmacotherapy implications:** Mechanism alignment with iloprost/teriparatide/BMAC/HBOT is thus highest at Type 1, partial at Type 2A, and negligible at Type 2B-3, which drives the Bain-Type-conditioned stratification of Section 5.7 and Section 8.5: a Bain Type 3 wrist is, by cartilage-state definition, a salvage problem regardless of Lichtman stage, whereas a Lichtman IIIA Type 1 wrist is the prime population for an iloprost/BMAC trial.

**Modality for trial baseline:** Diagnostic arthroscopy is the gold-standard cartilage assessment but adds an operative procedure to a non-operative trial and should be reserved for inconclusive imaging. High-resolution 3 T proton-density fat-suppressed MRI is the accepted non-invasive surrogate, with the proximal capitate the most error-prone surface. Trial baseline grading requires either arthroscopic or 3 T MRI Bain-equivalent grading by an experienced MSK-radiology reader.

**Inclusion–exclusion summary for any pharma-Kienböck protocol: Type 1 (and Type 2A, only with a joint-preserving surgical adjunct) is eligible**; Type 2B and Type 3 are ineligible.

### 5.4. Evidence by Skeletal Site

None of the published reports describe the lunate.

*Femoral head AVN and transient BMES*: *Aigner compared* five-day IV iloprost with core decompression in a prospective MRI-controlled study, reporting complete BME remission in essentially all iloprost-treated patients at three months, with pain and function comparable to surgery [82]. A subsequent 104-patient multicentre cohort confirmed durable improvement at twelve months. Disch reported sustained MRI resolution and clinical benefit in transient osteoporosis of the hip [79], Jäger 2008 provided a mechanistic synthesis [83], and Meizer reported the largest mixed-BMES series (n > 100) [84].

*Knee: SPONK and BME*: Mayerhoefer’s randomised oral-iloprost-vs-tramadol trial in knee BME demonstrated greater MRI BME resolution in the iloprost arm at three months, with analgesia comparable to tramadol [85]. Kirschbaum 2025 published a single-centre, prospective IV iloprost protocol for SPONK with stage-stratified MRI and PROM endpoints, which is methodologically the closest neighbour to a Kienböck pilot [80].

*Talus and other small bones*: Aigner’s 2001 *JBJS Br* case series described early talar AVN and BMES treated with IV iloprost with clinical and radiological improvement; this was the first translation of the hip protocol to non-weight-bearing small-bone ischaemia [86].

*Meta-analytic synthesis*: Zippelius 2022 pooled outcomes across femoral head, knee, talus and BMES, reporting aggregate VAS reduction ~3.3 cm and Harris Hip Score improvement ~24 points at three–six months with low SAE rates [32]: the headline translational signal, though it inherits the Vienna-network dominance (Section 2.5).

*Lunate/Kienböck*: No published cases of IV iloprost in Kienböck. A frequently mis-cited 2007 “Bartolomei” wrist report was confirmed on primary-source checking not to exist; the Vienna group has never published a Kienböck case. The Kienböck-specific iloprost evidence base is therefore empty.

### 5.5. The Translation Argument

The case is mechanistic and analogical, not direct. The drug’s mechanism targets precisely the pathophysiology accepted for Lichtman I–II and the residual BME/microvascular substrate of Stage IIIA, where iloprost is a surgical adjunct rather than primary therapy. Efficacy is consistent at biologically analogous sites (femoral head, knee, talus), including under randomised conditions and with MRI-defined endpoints. The absence of Kienböck-specific publications reflects publication and case-volume bias, not mechanistic failure.

### 5.6. Limitations and Caveats

Evidence at all bone sites is dominated by a single Vienna network, inherited by the Zippelius 2022 meta-analysis (Section 2.5) [32]. No placebo-controlled double-blind RCT of IV iloprost has been published at any skeletal site (Mayerhoefer 2007 used an active oral comparator [85]). Iloprost is off-label for all bone indications, and the five-day IV regimen requires inpatient or day-hospital infusion. SmPC adverse-event rates include headache 60–90%, flushing 40–60%, symptomatic orthostatic hypotension 20–30%, nausea/vomiting 20–40%, and prostacyclin-class jaw pain/trismus up to 10%, and the long-term safety of repeat cycles is unstudied.

**Regulatory geography.** IV iloprost (Ilomedin^®^) is nationally approved in several EU member states and by Swissmedic and the UK MHRA for vascular indications, but it has no central EMA or US FDA marketing authorisation; inhaled iloprost (Ventavis^®^) holds EMA central approval for PAH [81]. It is contraindicated in pregnancy (Category D per SmPC). Key interactions are additive hypotension with antihypertensives/vasodilators, synergistic vasodilation with PDE-5 inhibitors, and additive antiplatelet effect with anticoagulants/NSAIDs; there are no clinically meaningful CYP interactions.

### 5.7. Practical Clinical Recommendation

**Iloprost in Kienböck disease must be an adjunct or alternative to surgical management, not a substitute; established conservative and surgical management remain the standard of care.** The established surgical pathway (RSO, capitate-shortening osteotomy, VBG variants) remains primary for Lichtman IIIA and beyond [13]. Pharmacotherapy is mechanism-aligned only at Bain Type 1 and Type 2A (Section 5.3); Type 2B and Type 3 wrists are excluded.

Within those constraints, off-label IV iloprost may be considered in: (i) Lichtman I and II (pre-collapse: BME ± sclerosis without lunate collapse) with MRI-confirmed BME *(GRADE QoE Low, arguably Very Low; consistent benefit at femoral head, knee and talus with MRI endpoints and at least one positive knee RCT; no direct Kienböck data [80,82,85]);* (ii) Lichtman IIIA × Bain Type 1 or 2A, framed *strictly as an adjunct to joint-levelling surgery* (RSO/CSO/VBG; or proximal-pole-directed VBG/RSL fusion at Type 2A), not a substitute *(GRADE QoE Very Low; Section 4.2 argues against anti-resorptive efficacy beyond the pre-collapse window);* and (iii) Lichtman I/II patients in whom surgery is declined, delayed or contraindicated. Use requires multidisciplinary discussion and documented informed consent naming the absence of Kienböck-specific evidence.

An extrapolated protocol, offered for investigational use rather than as a treatment recommendation, is 20 µg iloprost in 500 mL saline IV over six hours daily for five days, body-weight-titrated within the licensed 0.5–2 ng/kg/min range, with per-infusion BP monitoring; dose, schedule and route are transposed from the Aigner protocol [82] without Kienböck-specific dose-finding (GRADE Very Low). Repeat cycles are used empirically but not formally studied. Outcomes comprise repeat MRI at three months, and PRWE, grip strength, and DASH at three, six, and twelve months.

## 6. Anabolic Agents and Other Adjuvants

Whereas anti-resorptive agents (Section 4) slow the catabolic limb of post-ischaemic remodelling, the agents reviewed here either drive new bone formation, modify the vascular and oxidative microenvironment, or supply cellular building blocks. Each agent is presented at the level of evidence that exists; GRADE QoE tags follow Section 2.4 and apply to Kienböck-specific use, not the strongest cross-site indication.

### 6.1. Teriparatide and Anabolic PTH Analogues

Teriparatide (recombinant PTH 1–34) and abaloparatide exploit the biology of intermittent PTH: subcutaneous pulses extend osteoblast lifespan and activate Wnt signalling, whereas continuous elevation is catabolic. Romosozumab, a humanised anti-sclerostin monoclonal antibody, acts upstream by releasing the Wnt brake. The pivotal Cosman FRAME trial established postmenopausal osteoporosis fracture-prevention efficacy [87], but romosozumab carries an FDA cardiovascular black-box warning within 12 months of MI/stroke, derived from the ARCH year-1 CV-event imbalance [88].

**MRONJ: the best-validated anabolic indication.** Sim 2020, a placebo-controlled trial of teriparatide-driven MRONJ healing [25], is the only RCT proving pharmacological reversal of established osteonecrosis at any skeletal site. Bashutski 2010 [55], Cheung and Seeman [89], and the 2022 AAOMS position paper [24] further support teriparatide as an adjunct for refractory MRONJ. GRADE Moderate for MRONJ; because its biology is jaw-specific, this evidence transfers only weakly to the lunate, supporting the anabolic principle rather than a lunate indication.

**Femoral head, knee, lunate:** Arai’s small retrospective ONFH comparison showed comparable sphericity preservation and a non-significant teriparatide trend versus alendronate [54] (Low for ONFH); Yokota 2025 adds modest post-osteotomy support [90]. Horikawa described three SPONK patients with pain resolution and stabilisation on teriparatide [91] (Very Low). No peer-reviewed Kienböck case has been published (Very Low, indirectness). The lunate is small, cancellous, and modest in necrotic volume, favouring pulse-PTH anabolism outpacing the resorptive front before collapse. Translation remains a reasonable hypothesis and the area most in need of a structured pilot.

**Regulatory status:** The FDA teriparatide osteosarcoma boxed warning and 24-month ceiling, based on rodent supratherapeutic dosing, were eliminated in November 2020 [92]; EMA followed in 2022 [93]. Retreatment is now permitted, making a 6–12-month course feasible for small-bone trabecular reorganisation. Adverse events include transient hypercalcaemia, orthostatic dizziness and dose-dependent ostealgia; contraindications include skeletal immaturity, prior irradiation, Paget disease, unexplained ALP elevation and skeletal malignancy. “Anabolic-first, anti-resorptive-after” sequencing has support from DATA-Switch [94]; the reverse sequence is inferior.

### 6.2. Denosumab

Denosumab (anti-RANKL monoclonal, Prolia^®^ 60 mg q6mo; Xgeva^®^ 120 mg q4w) has AVN evidence limited to isolated reports. The principal caution is post-discontinuation rebound: cessation without bisphosphonate consolidation can precipitate multiple vertebral fractures within months, a serious liability in young Kienböck patients. It requires no renal dose adjustment (an advantage where bisphosphonates are contraindicated); first-dose hypocalcaemia mandates calcium and vitamin D repletion. The lunate role remains speculative, an anti-resorptive niche already filled by bisphosphonates. GRADE: Very Low for Kienböck.

### 6.3. Statins

Statins inhibit HMG-CoA reductase upstream of the mevalonate pathway, which is also exploited by N-bisphosphonates; pleiotropic effects on endothelial NO, marrow adipogenesis, and BMP-2 expression underpin the literature on steroid-induced AVN prevention. Pritchett’s retrospective case–control study suggested that statin co-administration reduced ONFH in patients on high-dose corticosteroids [37]; Yang’s 2016 meta-analysis of preclinical models reinforced plausibility but remains animal-data-limited [95]. Most Kienböck patients are not steroid-driven, so direct translation is weak. When corticosteroids are unavoidable in a Kienböck patient, statin co-administration offers a low-cost risk-reduction strategy. GRADE Low for steroid-AVN prevention; Very Low for non-steroid-exposed Kienböck.

### 6.4. Hyperbaric Oxygen Therapy

HBOT drives oxygen by diffusion into the ischaemic penumbra, reactivating osteoblastic activity and suppressing osteoclastic activity. Camporesi’s sham-controlled RCT in early ONFH [96] remains the highest-quality AVN evidence, with benefit at one and two years; the MRONJ literature treats HBOT as an established adjunct. In the wrist, Cracchiolo reported a single Lichtman IIIC Kienböck case treated with HBOT alongside surgical decompression, with radiographic stabilisation at follow-up [18]. Typical protocols run at 2.0–2.5 ATA for 60–90 min over 30–40 sessions; cost and chamber access are practical barriers. Mechanistically, it complements iloprost: more oxygen per unit perfusion versus more perfusion per unit oxygen. GRADE: Low for Kienböck (downgraded for indirectness); an investigational adjunct only.

### 6.5. Anticoagulants

Glueck demonstrated over-representation of inherited thrombophilias in idiopathic ONFH and proposed enoxaparin prophylaxis [43]. Kienböck’s disease is not primarily a thrombotic disorder; the only defensible empirical indication is for a Kienböck patient with an independently documented thrombophilic diathesis, in whom standard prophylaxis already applies. GRADE: Very Low.

### 6.6. Vitamin D and Calcium

Vitamin D repletion (25(OH)D ≥ 30 ng/mL) and elemental calcium (1000–1200 mg/d) underpin every other agent reviewed and are required by bisphosphonate, denosumab, and teriparatide labels before initiation; a single 25(OH)D and corrected-calcium measurement belongs in the standard work-up before any Section 4, Section 5 and Section 6 pharmacotherapy. Not GRADE-rated (preconditional).

### 6.7. Pentoxifylline and Tocopherol (PENTO Protocol)

The PENTO regimen (pentoxifylline 400 mg b.i.d. plus vitamin E 400–1000 IU/d) couples a haemorheological/vasoactive arm with an antioxidant arm and remains a MRONJ-adjunct mainstay popularised by Delanian [97]; both arms are theoretically relevant to the necrotic lunate but unstudied. Safety paradox: the tocopherol component sits above the ≥150 IU/d all-cause-mortality signal in Miller 2005 [98]; the net benefit is judged to outweigh harm for short-course exposure, but any Kienböck protocol should pre-specify duration. Pentoxifylline modestly potentiates anticoagulants, and INR monitoring is required on warfarin. GRADE: Very Low for Kienböck.

### 6.8. Mesenchymal Stromal Cells and Bone Marrow Concentrate

**Regulatory dividing line (operationally decisive for any Kienböck cellular-therapy protocol): Same-session centrifuge-prepared BMAC is not an ATMP (Reg 1394/2007 Art 2(1)(b) plus Annex I); Directive 2004/23/EC and surgical-practice provisions apply.** Culture-expanded autologous MSC therapy is a full ATMP requiring a GMP facility, IMPD, CAT classification, and CTA under EU Reg 536/2014; a registered autologous MSC trial used this pathway [99]. Allogeneic products are a separate ATMP class; no Kienböck use to date.

**Evidence base.** Hernigou’s ONFH BMAC cohort remains the largest single-centre AVN cellular-therapy experience [58], consistently reporting reduced collapse and arthroplasty when delivered pre-collapse. The most Kienböck-relevant cellular therapy is the registered five-patient cultured autologous MSC series combined with core decompression and vascularised bone graft, the only registered pharma-adjacent Kienböck intervention [17]. Ogawa combined BMA, LIPUS, and external fixation [20], an attribution confound; a minimally manipulated BMAC arm is materially more tractable than a cultured-MSC-only Phase II. GRADE: Very Low for Kienböck; both remain investigational and appropriate only within a registered trial.

### 6.9. Local Application of Bone Morphogenetic Protein

rhBMP-2 (FDA-approved for lumbar spinal fusion only) and rhBMP-7 (withdrawn from major markets) are off-label for AVN; heterotopic ossification and oncogenic signalling concerns disfavour Kienböck use; the sparse literature is confined to combination protocols with no Kienböck-specific reports, and BMP appears here for completeness only. GRADE: Very Low.

### 6.10. Synthesis

Teriparatide has the strongest cross-site “anabolic-reverses-osteonecrosis” pedigree (jaw Moderate; ONFH/SPONK Low/Very Low) and is the most defensible candidate for a Kienböck investigational protocol despite the absence of any published lunate case; the regulatory headwind has lifted (Section 6.1 [92,93]). HBOT and PENTO are well-tolerated, mechanistically complementary adjuncts that could layer onto a vasoactive backbone (Section 5). Autologous cellular therapy is the only modality with a registered Kienböck-specific trial; the BMAC-versus-ATMP dividing line (Section 6.8) is operationally decisive for any Phase II [99]. Denosumab, statins outside the steroid-exposed subgroup, anticoagulants, romosozumab, and BMP occupy narrow or speculative niches. Consistent with the collapse-boundary hierarchy (Section 4.2), pharmacological options are first-line candidates at Lichtman I and II (pre-collapse) and at most surgical adjuncts from IIIA onward, with no pharmacological modification of the surgical algorithm from IIIB to IV.

## 7. Site-Specific Evidence: A Cross-Cutting Comparison

Where Section 4, Section 5 and Section 6 move by drug class, Section 7 takes the orthogonal site-by-site cut. The drug × site evidence matrix (Table 1) states the field’s central problem visually: empty carpal columns against populated extra-carpal ones.

**Site summary (anchored on Table 1).** The femoral head is the reference site; ARCO 2019 is operationally cognate with Lichtman I–IV, and at least four drug classes show benefit pre-collapse before losing it post-subchondral fracture (collapse boundary, Section 4.2). At the jaw, drug effects split by mechanism, not by site: the bisphosphonate paradox does not generalise to the microbially sealed intra-articular lunate (Section 4.4), whereas the teriparatide reversal (Sim 2020 [25]) and PENTO act through site- and microbe-agnostic mechanisms. Knee SPONK is the closest structural comparator, and the Meier 2014 null [26] is the cautionary signal every Kienböck bisphosphonate trial must mitigate. Talus AVN, loaded but not weight-bearing and with a watershed end-arterial supply, offers the defensible bridgehead for extending the Vienna iloprost protocol to the wrist. Humeral head and Freiberg/Kümmell add no leverage, though Freiberg-Köhler carries case-level bisphosphonate observations within Yoshimura’s 2024 algorithm [100].

**Cross-site discordances.** (i) Bisphosphonates treat hip AVN pre-collapse yet *cause* jaw AVN through mechanisms not operative at the lunate (Section 4.4). (ii) Within-site disagreement (Lai vs. Chen; Jureus vs. Meier) is best reconciled by stage-mix heterogeneity. (iii) Iloprost has a consistent positive signal at five sites but no Kienböck data at all. (iv) Teriparatide reverses MRONJ under randomised conditions [25] but remains untested at the lunate. The integrative read is mechanistic and stage-stratified, not site-uniform.

**Surgical-pathway primacy.** At every extra-carpal site where pharmacotherapy has a positive signal, it sits alongside an established surgical pathway, not in place of one, and translation to Kienböck preserves this hierarchy. Pharmacotherapy is a defensible first-line candidate alongside conservative management at Lichtman I–II, at most an adjunct to stage-appropriate joint-preserving surgery at IIIA, and has no demonstrated disease-modifying role at IIIB–IV, where the surgical pathway remains primary. The Stage-IIIA boundary marks the field’s most active disagreement; the extra-carpal record does not license pharmacotherapy to displace RSO or VBG as the standard for a working-age Stage-IIIA patient.

## 8. Translation to the Lunate and the Carpal Avascular Necrosis Family

### 8.1. The Translation Problem

The evidence base is unevenly distributed: substantial for the femoral head, mature for the jaw, accumulating for SPONK, and essentially empty for the lunate, where the dedicated record reduces to the roughly twenty patient-level observations of Section 1 [15,16,17,18,19,20], none randomised, Lichtman-stratified or guideline-threshold. Translation from adjacent sites is therefore the only realistic path until a Kienböck-specific randomised programme can be funded, but this approach must itself be justified.

### 8.2. A Four-Axis Translation Framework

The framework operationalises three established traditions: drug-repurposing methodology [101] (mechanistic plausibility, PK/safety, regulatory tractability, bridging evidence); the NIH T0–T4 translational-science spectrum [102] (KD sits at T1–T2); and the REWARD initiative on avoidable waste [103]. The Lichtman, ARCO, Steinberg, and Herbert–Lanzetta cross-staging convention used throughout is the present authors’ working analytical alignment, not formally derived from a primary-source concordance. The framework is an unvalidated heuristic meant to structure reasoning and generate hypotheses: its axes have not been tested against outcomes, and it should not by itself justify clinical prescribing.

**Axis 1: Mechanistic alignment:** Three Kienböck-operative pathways: SIF (Yamamoto-Bullough SPONK reframe); microvascular ischaemia in a watershed bed [104]; and revascularisation-phase resorptive collapse (Aspenberg monkey-lunate hypothesis, Section 3.6) [14]. Anti-resorptives engage pathways 1 and 3; vasoactive agents and HBOT engage 2; anabolic agents engage 1 by a different lever. Axis 2: Anatomic concordance: SPONK and Preiser score highest (small, cartilage-covered, intra-articular, locally loaded); talus and humeral head intermediate; femoral head most evidence-rich but anatomically remote [105]. Axis 3: Stage applicability: At *Lichtman I–II (ARCO I–II, SPONK pre-collapse)* pharmacotherapy retains first-line rationale; at *Lichtman IIIA (early ARCO III, post-fracture SPONK)* it is adjunct to joint-preserving surgery only, since the collapse boundary (Section 4.2) and the Meier 2014 SPONK null [26] caution that anti-resorptive efficacy is unlikely to survive it, while a residual vasoactive/anti-oedema rationale persists; at *Lichtman IIIB, IIIC and IV* surgery dominates. The Schmitt perfusion pattern and the Bain and Begg cartilage type (Section 5.3, refs. [11,12]) gate candidacy independently of osseous stage: pharmacotherapy retains rationale only where Lichtman I–IIIA, Schmitt A or B and Bain Type 1 or 2A converge. Axis 4: Risk–benefit at the lunate: Jaw-specific mechanisms (Section 3.5) have no lunate analogue [60,62,63]; iloprost’s hypotension and headache are site-independent; the romosozumab CV BBW remains while the teriparatide warnings were eliminated (Section 6.1) [92,93]; cell-therapy and deferoxamine carry site-independent regulatory complexity.

**Schmitt perfusion-MRI and imaging-endpoint translatability.** The four axes identify which drug class fits which biology, but not which patient has a substrate the drug can act on; the lunate-specific resolution is the Schmitt perfusion-MRI classification [106], graded on dynamic gadolinium-enhanced fat-saturated T1 sequencing: Schmitt A (diffuse homogeneous enhancement, marrow viable, revascularisation-amenable); Schmitt B (partial enhancement with geographic non-enhancing zones, mixed substrate); Schmitt C (no enhancement, established irreversible necrosis). Vasoactive, HIF-axis, oxygen-supply, and cellular-substrate-dependent interventions all require enhancement-evidenced viability (Schmitt A or early B) at entry; enrolling Schmitt C disease reproduces at the lunate the stage-mix dilution invoked in Section 4.2 [21,22]. The Müller 2019 DCE-MRI/histopathology correlation [107] shows that enhancement geography, not amplitude, distinguishes repair from necrosis. Extra-carpal evidence therefore translates only insofar as it uses a modality deployable at the lunate (a provisional fifth axis): MRONJ-teriparatide (CT-based) is down-weighted, whereas SPONK/talus-iloprost (MRI BME [80,86]) is structurally translatable. Both Schmitt and the Bain articular-surface companion (Section 5.3) [11] are accordingly mandatory entry-criterion descriptors for any future Kienböck trial.

### 8.3. Application Across Drug Classes

The strongest case overall is iloprost, converging on every axis except for direct lunate data, which motivates the manuscript’s principal pilot proposal. The strongest class with any Kienböck-patient data is bisphosphonates [15], where the Lai/Chen divergence and the Meier 2014 negative SPONK RCT [26] oblige any lunate extension to be strictly Lichtman I–II stratified.

### 8.4. The Carpal Avascular Necrosis Family

The dominant frame treats the lunate as *sui generis*, comparing it to the much larger, biomechanically remote femoral head and almost never to its closest anatomic neighbours. We propose situating it within a carpal AVN family comprising lunate (Kienböck) [2,3], scaphoid (Preiser) [28], capitate (idiopathic), hamate [29] and triquetrum. This is a proposed research grouping, not a nosological family or a claim of clinical equivalence: the carpal bones differ in vascular anatomy and aetiology, and this heterogeneity limits how far evidence can be borrowed.

The argument is methodological: the commonalities support a unified basket-trial approach for proof-of-concept studies, even though stage-specific surgical management remains site-specific. Cohesion rests on five dimensions: anatomy (intra-articular, centimetre-scale, cartilage-covered, small-bone trabecular geometry [105]); vascular architecture (end-arterial/watershed [104]); idiopathic dominance (outside post-traumatic scaphoid non-union); natural history (BME to sclerosis to fragmentation to collapse); and evidence-base depth (the Preiser literature [28] is even thinner than that for Kienböck’s disease).

Each family member is too rare for stand-alone randomised programmes: Preiser, ~170 reported cases [28]; idiopathic capitate AVN, <30; hamate, ~12 [29]; triquetrum, a handful since 2005. Carpal AVN is therefore a defensible hypothesis-generating basket-trial candidate, though not an archetypal one in the strict oncologic-exchangeability sense: the scaphoid’s non-exchangeable vascular substrates (Gelberman & Menon 1980 [104]) and Preiser’s idiopathic-versus-post-traumatic dichotomy (Kalainov 2003 [108]) are real axes of heterogeneity. Any basket protocol must pre-specify the exchangeability assumption, stratify the scaphoid basket by Kalainov type, and treat Bayesian borrowing as a sensitivity analysis rather than primary inference. One configuration remains defensible: a single multi-centre European protocol with Lichtman I–II Kienböck as the primary stratum (plus an optional Stage IIIA adjunct-to-surgery sub-stratum), Herbert–Lanzetta I–II true-idiopathic Preiser as the parallel stratum, rarer sites enrolled exploratorily, post-traumatic Preiser routed to a documented-trauma registry stratum, and a shared ICHOM-aligned outcome set [52]. This construct, for proof-of-concept hypothesis-generating work rather than a strict-exchangeability basket, is the principal conceptual contribution of the review.

### 8.5. Cautious Practice Recommendations for Today

**Established conservative management (immobilisation, hand therapy, analgesia) and, from Lichtman IIIA onward, established surgery (RSO, capitate-shortening osteotomy, VBG) remain the standard of care; the options below are investigational, apply only to Lichtman I–II or to IIIA-and-later patients in whom surgery is contraindicated, declined, or deferred, and should never delay stage-appropriate surgery. They fall into three tiers: reasonable investigational options within a trial or a documented off-label pathway (chiefly iloprost, and stage-limited bisphosphonates, in Lichtman I-II); options with insufficient evidence to recommend even off-label (teriparatide, HBOT, cellular therapy); and options not recommended outside a trial at any stage (denosumab, romosozumab, BMP, and pharmacotherapy of any kind as a substitute for indicated surgery from Lichtman IIIA onward).**

**A conservative backbone applies across all strata:** volar/circumferential neutral-wrist orthosis for 6–12 weeks at trial entry, hand therapy 1–2× weekly, and staged return-to-work with modified duty at weeks 4–8 and full duty at weeks 12–16, conditional on PRWE Δ ≥ MCID (11.5 per Walenkamp 2015 [51]).

**In Lichtman I or II (pre-collapse, MRI-confirmed BME),** IV iloprost (Vienna protocol, Section 5.7) is the single most defensible option to investigate: the mechanistic case across BME-equivalent talus [86], knee [80,85] and hip [79,82] sites is strong, but Kienböck-specific evidence is zero, and the patient must be told this is an extrapolation. The Agarwala combination protocol (Section 4.6 [15]) is the only one with prospective Kienböck data, and is particularly useful with concurrent steroids, where statins have observational support [37]; HBOT may be added where access permits [18,19], and MSC/BMAC retain Kienböck signal [17,20] but belong within a clinical-trial framework (Section 6.8).

**From Lichtman IIIA, the stage-specific recommendations follow Section 9.3: pharmacotherapy is a documented, consented adjunct to stage-appropriate surgery only in the surgically fit patient. Two clinical-decision branches operate in parallel: cartilage state (Bain & Begg articular type, Section 5.3, refs. [11,12]) and ulnar variance. Bain Type 1 or 2A wrists are joint-preserving candidates and the prime stratum for any pharma-adjunct protocol (RSO for negative variance; capitate-shortening osteotomy for neutral or positive; VBG variants in selected cases); Type 2A wrists are candidates for proximal-pole-directed reconstruction with pharma as defensible perioperative adjunct; Type 2B and Type 3 wrists are operationally salvage problems regardless of stage.** Pharmacotherapy is adjunct only at Bain Type 1/2A (perioperative bisphosphonate per the Aspenberg hypothesis [14], or post-operative iloprost for residual BME, Section 5.3): it must not substitute for or delay the structural intervention without explicit shared decision-making, is not indicated at Bain Type 2B/3, and requires consent naming the absence of Kienböck-specific evidence.

**Lichtman Stage IIIA in the surgically unfit/declining/deferring patient.** Pharmacotherapy assumes a larger role as the principal non-operative alternative; the same iloprost or Agarwala options apply, with explicit acknowledgement that the natural history of Stage IIIA without surgery is one of progression to Stage IIIB/IV in the majority, and that pharmacological options have not been shown to alter the trajectory.

**Lichtman Stage IIIB and IV (carpal collapse, secondary arthrosis).** The pharmacological approach recedes, and the surgical pathway (PRC, four-corner fusion, total wrist arthroplasty) takes over, with only a narrow perioperative HBOT role (Cracchiolo IIIC [18], Figueira IIIA [19]).

### 8.6. What Should Not Be Done

First, dosing should not be extrapolated unmodified from femoral-head trials without a Kienböck dose-finding pilot, since the lunate is two orders of magnitude smaller and intraosseous exposure likely differs. Second, bisphosphonates should not be commenced without strict Lichtman-stage confirmation: Meier 2014 [26] is the most lunate-comparable RCT and shows that pharmacotherapy delivered after the structural fate is sealed does not modify outcome. Third, teriparatide should remain a trial-setting agent: the only randomised proof of reversal of established osteonecrosis comes from MRONJ (Sim 2020, Section 6.1) [25], whose biology does not transfer to the lunate. Fourth, patients must not be promised efficacy: each intervention is mechanism-plausible but evidence-thin; the Section 8.4 carpal AVN family programme is the route by which evidence might eventually be assembled.

## 9. Evidence Gaps and Conclusions

### 9.1. Evidence Gaps

Kienböck pharmacotherapy rests almost entirely on inference from extra-carpal sites, with no disease-specific randomised evidence for any class [2,3]. The Section 3, Section 4, Section 5, Section 6, Section 7 and Section 8 synthesis reveals a ten-item gap inventory. (1) No Kienböck-specific RCT exists for any drug class; every agent has been studied in KD only in case reports, single-arm series, or retrospective cohorts < 50 patients [15,21,22]. (2) No Lichtman-stage-stratified pharmacotherapy data exist. (3) No comparative effectiveness is established for mechanistically plausible combinations. (4) No PROM/MCID has been anchored to pharma-Kienböck cohorts (PRWE MCID ≈ 11.5 [51] as working anchor). (5) No follow-up beyond 5 years exists [15,21,22]. (6) No carpal AVN family comparative safety dataset exists. (7) No formal Kienböck dose-finding exists; all schedules are extrapolated [52]. (8) A publication gap surrounds the Vienna iloprost-Kienböck experience [80,82]. (9) No active pharma-Kienböck registry exists beyond a single small MSC series [17]. (10) No COMET-endorsed Kienböck core-outcome set exists; in the interim, the ICHOM Standard Set for Hand and Wrist Conditions anchors outcome assessment, with a four-level DOOR ordering from BME resolution without surgery and PRWE Δ ≥ MCID down to surgery or Lichtman progression.

### 9.2. Conclusions

For a pharmacology readership, the lunate is an unusually instructive case of drug repositioning in a rare, thin-evidence indication: a single drug class (iloprost) survives all four translation axes intact, with the most directly transposable protocol templates (Aigner 2005 [82]; Kirschbaum 2025 [80]). Bisphosphonates carry the largest absolute evidence base, but with unresolved Lai-vs-Chen heterogeneity (Section 4.2) [21,22]. Anabolic agents (teriparatide, abaloparatide) and biologics (denosumab, anti-sclerostin) are theoretically attractive but largely untested. No drug class is ready for unqualified recommendation in routine practice; all current-practice statements carry Low-to-Very-Low QoE by GRADE [31].

The review contributes two outputs. The carpal AVN family concept (Section 8.4) provides a cohesive analytic block for trial design in conditions that are individually too rare for solo RCTs, and the four-axis heuristic (Section 8.2) supplies an explicit rubric for evaluating imported extra-carpal evidence. Both contributions are exploratory: the four-axis framework is an unvalidated heuristic and the proposed carpal grouping is a hypothesis for trial design rather than a validated construct. We do not recommend routine pharmacological treatment of Kienböck disease; the central message is that Kienböck-specific evidence does not yet exist, and that established conservative and surgical management remain the standard of care.

### 9.3. What Should Clinicians Do Today?

**Lichtman I–II.** A pharmacological adjunct (iloprost or stage-stratified bisphosphonate) may be considered within a trial or a documented off-label pathway, with explicit disclosure that all recommendations rest on extrapolation. GRADE: Very Low to Low; conditional recommendation.

**Lichtman IIIA.** Contested stratum: Surgery (RSO, capitate-shortening, VBG variants; diagnostic arthroscopy with Bain & Begg assessment where indicated) has prospective comparative decade-long follow-up and remains standard of care. Pharma is appropriate only as (a) an adjunct to surgery, (b) a bridge in patients declining or not currently candidates, or (c) within a registered trial. GRADE: Very Low; conditional recommendation against pure pharma-only at IIIA.

**Lichtman IIIB–IV.** Surgery remains the primary treatment: PRC, partial wrist fusion, and salvage arthroplasty. Pharmacological evidence does not modify the surgical algorithm.

Across all stages, patients must be informed that pharma-KD recommendations rest on extrapolation, not Kienböck-specific RCT evidence, and, where a registry or trial is available, enrolment should be the default.

## Figures and Tables

**Table 1:** Drug × site evidence matrix (summary): Cells give the OCEBM per-study evidence level (L1–L5; see Section 2.5) and direction of effect (+ positive, − negative/harm, ± mixed, ? insufficient; — no published evidence identified at this drug × site combination); supporting citations are given in Section 4, Section 5, Section 6 and Section 7. (In the lunate column, every positive signal derives from case reports or series below ten patients and is preliminary; evidence level, direction of effect, and applicability to Kienböck are distinct dimensions).

Drug Class	Femoral Head	Humeral Head	Knee (SPONK)	Talus	MRONJ (Jaw)	Lunate (Kienböck)	Scaphoid (Preiser)	Capitate	Other Carpal
Aminobisphosphonates	L1 (±)	L4 (+)	L1 (−); L4 (+)	L4 (+)	L5–L4 causative (−)	L4 (+)	L5 (?)	—	L5 Freiberg (?)
Iloprost (IV/oral)	L2 (+)	L5 (?)	L2 (+); L4 (+)	L4 (+)	—	—	—	—	—
Teriparatide (PTH 1–34)	L3 (+)	—	L5 (+)	—	L1 (+); L4 (+)	—	—	—	Distal radius L1; pelvic L2; sacral L4
Abaloparatide	—	—	—	—	—	—	—	—	OP only
Romosozumab	—	—	—	—	L5 causative (−)	—	—	—	OP L1 (+)
Denosumab	L3 (+)	—	—	—	L4 causative (−)	—	—	—	—
Statins	L3 (±)	—	—	—	—	—	—	—	CV prevention only
HBOT	L1 (+)	—	L3 (+)	L5	L2 (±)	L5 (+)	—	—	—
Anticoagulants	L2 (+)	—	—	—	—	L5 (?)	—	—	—
Vitamin D/Calcium	L5 (+)	—	L5	—	L4 (+)	—	—	—	—
Stem cells/BMA/BMAC	L1–L2 (+)	—	—	—	—	L4 (+)	—	—	—
BMP-2/BMP-7	L4 (+)	—	—	—	—	—	L3 (+)	—	—
PENTO/PENTOCLO	L5 (?)	—	—	—	L1 (+)	—	—	—	—
Deferoxamine (iron chelator)	—	—	—	—	—	L4 (+)	—	—	—
PDE5 (sildenafil)	L5 (+)	—	—	—	—	—	—	—	—
Antioxidants (Vit E, NAC, CoQ10, resveratrol, melatonin, curcumin, α-LA)	L5 preclinical + L1 safety (−)	—	—	—	—	—	—	—	—
NSAIDs/Analgesics	Symptomatic	Symptomatic	Standard adjunct	Symptomatic	Symptomatic	Symptomatic	Symptomatic	Symptomatic	Symptomatic

**Table 2:** Four-axis translation framework (summary).

Axis	Question	Application to the Lunate
1. Mechanistic alignment	Does the extra-carpal evidence share the SIF/microvascular-ischaemia/hypercoagulability pathway dominant at the lunate?	High for SPONK (SIF); variable for ONFH (multipath); low for MRONJ (alveolar-specific).
2. Anatomic concordance	Is the donor site comparable in size, end-arterial supply, weight-bearing status, and microbial exposure?	Highest for other carpal AVN; moderate for SPONK; low for ONFH (size) and MRONJ (microbial).
3. Lichtman × Schmitt × Bain stage applicability	Does donor evidence map onto early (I–II), intermediate (IIIA–IIIB), or salvage stages across all three axes (osseous + perfusion + cartilage)?	Iloprost: I–IIIA × Schmitt A/B × Bain Type 1 or 2A; bisphosphonate: II–IIIA timing-dependent × Schmitt A/B × Bain Type 1 or 2A; teriparatide: post-revascularisation × Bain Type 1 (cartilage substrate intact).
4. Lunate-specific risk–benefit	Are off-target risks at the lunate procedurally manageable and proportionate to expected benefit in a rare disease?	MRONJ-paradox resolved with procedural-breach caveat (Section 3.5); rebound on denosumab discontinuation; romosozumab CV BBW; teriparatide BBW eliminated.

## Data Availability

No new data were created in this study. Data sharing is not applicable to this article. The synthesis of all findings is described in the manuscript itself.
